# Recent Progress on the Localization of PLK1 to the Kinetochore and Its Role in Mitosis

**DOI:** 10.3390/ijms23095252

**Published:** 2022-05-08

**Authors:** Taekyung Kim

**Affiliations:** Department of Biology Education, Pusan National University, 2, Busandaehak-ro 63beon-gil, Geumjeong-gu, Busan 46241, Korea; taekim@pusan.ac.kr; Tel.: +82-51-510-2693; Fax: +82-51-514-8576

**Keywords:** PLK1 kinase, kinetochore, spindle assembly checkpoint, chromosome segregation, PP1, PP2A, cell cycle

## Abstract

The accurate distribution of the replicated genome during cell division is essential for cell survival and healthy organismal development. Errors in this process have catastrophic consequences, such as birth defects and aneuploidy, a hallmark of cancer cells. PLK1 is one of the master kinases in mitosis and has multiple functions, including mitotic entry, chromosome segregation, spindle assembly checkpoint, and cytokinesis. To dissect the role of PLK1 in mitosis, it is important to understand how PLK1 localizes in the specific region in cells. PLK1 localizes at the kinetochore and is essential in spindle assembly checkpoint and chromosome segregation. However, how PLK1 localizes at the kinetochore remains elusive. Here, we review the recent literature on the kinetochore recruitment mechanisms of PLK1 and its roles in spindle assembly checkpoint and attachment between kinetochores and spindle microtubules. Together, this review provides an overview of how the local distribution of PLK1 could regulate major pathways in mitosis.

## 1. Introduction

During mitosis, it is critical for cells to correctly segregate duplicated genomes into daughter cells. Any errors arising in this process can cause aneuploidy observed in most cancer cells and may lead to birth defects [1,2]. Therefore, it is important to understand how chromosomes are accurately segregated during cell division.

A major component of the machinery required for this process is the kinetochore, a large multiprotein complex that mediates the attachment of chromosomes to spindle microtubule ends. In addition, kinetochores serve as a signaling platform for monitoring errors in attachment and directing progression through the cell cycle. When kinetochores are not attached to spindle microtubules, they delay the progression of anaphase by activating the spindle assembly checkpoint (SAC) to provide sufficient time for the interaction between kinetochores and microtubules. The SAC inhibits activation of the anaphase-promoting complex/cyclosome (APC/C), a large E3 ubiquitin ligase required for sister chromatid separation and execution of the mitotic exit [3,4].

An essential group of mitotic kinases, including CDK1, PLK1, Aurora B, Mps1, and Bub1, and the counteracting phosphatases PP1 and PP2A, are thought to regulate kinetochore assembly and chromosome segregation [5,6]. Polo-like kinase 1 (PLK1), a well-conserved mitotic serine/threonine kinase, is an essential mitotic kinase that localizes to kinetochores, centrosomes, and the central spindle and midbody. It plays important roles in mitotic progression, centrosome assembly, chromosome segregation, spindle elongation, and cytokinesis [7,8,9].

In addition, PLK1 has also been shown to be overexpressed in various types of cancers, including breast cancer, rectal cancer, colorectal cancer, pancreatic cancer, ovarian cancer, and lung cancer [10,11,12]. Overexpression of PLK1 in cancer cells is also associated with poor prognoses [10,11,13,14,15,16]. In fact, many different PLK1 inhibitors have been developed, and more than 10 PLK1 inhibitors are commercially available. Furthermore, some of the PLK1 inhibitors are in clinical trials and have demonstrated promising results in the treatment of many types of cancer [10,13].

However, it is currently unclear why overexpression of PLK1 causes cancer or is a consequence of the overproliferation of cancer cells [17]. If PLK1 is an oncogene, identifying which pathways involve PLK1 and cause carcinogenesis will be critical in developing therapeutic strategies. For example, chromosomal instability is a common feature in cancer cells and can be caused by chromosome missegregation in mitosis [1]. Therefore, elucidating how PLK1 regulates mitosis is an important question to be answered.

Because PLK1 has many diverse functions in mitosis, it is crucial to understand how PLK1 localizes at specific regions during mitosis, which will allow the dissection of the role of PLK1. PLK1 plays a critical role in SAC and chromosome alignment, and many candidates were suggested to recruit PLK1 to the kinetochore. However, the component essential for kinetochore recruitment of PLK1 remains unclear. Here, we first describe the structure and activation mechanism of PLK1 and then elaborate on how PLK1 localizes at the kinetochore. Finally, we discuss the role of PLK1 in SAC, chromosome alignment, and microtubule attachment.

## 2. Molecular Properties and the Activation of PLK1

PLK1 was initially discovered via genetic screening in budding yeast, in which the temperature mutation failed to progress through the cell cycle. The name Polo-like kinase (Plk) originated from a discovery in *Drosophila*, where the mutation of the gene resulted in abnormal spindle poles. PLK1 is well conserved among species, and almost all eukaryotes, except plants, have at least one form of the PLK family [18,19,20,21]. While flies, budding yeast, and fission yeast only have a single PLK family member, humans, mice, frogs, and worms have multiple PLK family members [22,23]. In humans, there are five PLKs, from PLK1 to PLK5, and they have little overlap in their localization and function, despite some structural similarity [8,22]. PLK2 is required for centrosome maturation and centriole duplication in the S phase, and PLK3 promotes DNA replication during the G1/S transition and is involved in the stress response pathways [24,25,26,27,28,29,30,31]. PLK4 plays a role in centriole duplication in the S phase, and PLK5 functions in neuron differentiation [31,32,33,34]. PLK1 is mainly expressed in late G2 and M phases and plays critical roles in the regulation of mitosis and cytokinesis [22,31,35,36,37].

**Figure 1:**
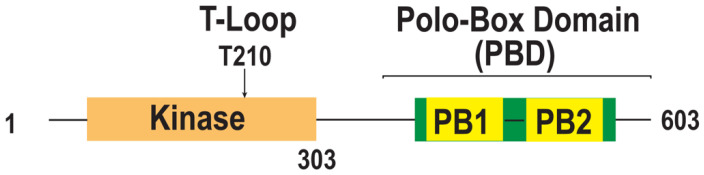
Domain organization of PLK1. A schematic diagram of human PLK1 protein and its domain organization. PLK1 contains the serine/threonine kinase domain (KD: orange), the polo-box domain (PBD: green), and the linker between two domains. The PBD consists of polo-box 1 and 2 (PB1 and PB2: yellow). PLK1 activation requires the phosphorylation of conserved Thr residues within the T-Loop.

PLKs are defined by the presence of an N-terminal catalytic Ser/Thr kinase domain and a C-terminal polo-box domain (PBD) that phosphorylate target proteins and control PLK1 kinase activity and localization, respectively [7,8,9,22]. In humans, kinase domains are highly conserved among all PLKs, except for PLK5, which lacks the kinase domain [22,23,33,38]. In contrast, the PBD region, which consists of two polo-box (PB) motifs, intervening residues, and a part of the linker between the kinase domain and PB1, is less well conserved [23] (Figure 1).

Among all PLK family members, PLK1 is the most highly conserved. PLK1 has also been the most extensively studied because of its importance in cell division. The kinase domain of PLK1 phosphorylates Ser or Thr of substrates that contain the consensus motif [D/N/E/Y]-[X]-S/T-[F/Φ; no P]-[Φ/X] [9,23]. Moreover, the kinase domain contains a T-loop, also called an activation loop, which allows PLK1 to convert ATP to ADP, transferring the phosphate group to PLK downstream phosphorylation substrates [39]. The PBD is required for localization at the target region, such as the centrosome, centromere, and the kinetochore at prometaphase and metaphase, and the central spindle and midbody at anaphase [40,41,42,43]. The expression of the kinase domain alone is not sufficient for PLK1 localization at the centromere and the kinetochore, and the expression of the PBD alone localizes strongly at the centromere and weakly at the kinetochore [44]. PBD recognizes the S-[pS/pT]-[P/X] motif of the target protein, and when X is proline, proline-directed kinases, including the Cdk1 kinase, can phosphorylate Ser or Thr [45,46]. Phosphorylation of the preceding Ser or Thr primes the motif, thereby generating a binding site for PBD docking [9,42]. Alternatively, when the S-[pS/pT]-[P/X] motif is phosphorylated by PLK1 itself, known as self-priming, PBD can interact with PLK1 [42].

Furthermore, PBD is required to control kinase activity. PBD docking partially activates Plk1 by inducing conformational changes [47]. However, to fully activate PLK1 and relieve the intramolecular interaction between the kinase domain and PBD, which stabilizes the inactive conformation of PLK1, phosphorylation of the T-loop in the kinase domain is required [40,47,48,49,50]. The T-loop of the kinase domain can be phosphorylated by Bora, and Aurora A and B, which localize at the centrosomes and kinetochore, respectively [49,50,51,52,53].

## 3. Function of PLK1 in Mitosis

PLK1 is a master mitotic kinase with diverse and important functions in mitosis, including mitotic entry, a G2/M checkpoint, centrosome assembly, chromosome segregation, a spindle assembly checkpoint, and cytokinesis [54,55,56,57,58,59,60,61,62,63,64,65,66,67]. PLK1 expression levels start to increase at the G2 phase and peak in the metaphase of the M phase. Upon completion of mitosis, PLK1 levels sharply decrease [35,36,37].

PLK1 is essential for mitotic entry and exit. Mitotic entry is controlled by CDK/cyclin B complex activity, which is regulated by phosphorylation. PLK1 activates Cdc25 phosphatase, which in turn inactivates the Wee1 and dephosphorylates CDK/cyclin B, leading to the activation of CDK/cyclin B [68,69,70,71,72]. In addition, PLK1 promotes Wee1 degradation by phosphorylation, which decreases phosphorylation of CDK/cyclin B by Wee1 kinase [73]. PLK1 is also involved in the activation of transcription factor Forkhead Box M1 (FoxM1), which allows the expression of the genes involved in the G2/M transition [74]. Additionally, PLK1 promotes activation of the anaphase-promoting complex (APC/C) and the degradation of the APC/C inhibitor Emi1 to exit mitosis and enter cytokinesis [75,76,77,78,79].

PLK1 also contributes to centriole duplication and centrosome maturation. Other PLK family members, PLK2 and PLK4, also localize to the centrosomes, but the function of PLK1 in centrosome maturation is not redundant with that of PLK2 or PLK4. During the S and M phases, PLK1 promotes centrosome maturation by phosphorylating several centriole proteins and nucleation factors, including pericentrin, cnn, Nlp, Kizuna, and γ-tubulin [80,81,82,83,84]. In addition, PLK1 allows centriole disengagement, leading to centriole duplication [85]. Interestingly, PLK1 was also suggested to contribute to DNA replication during the S phase [86].

During M phases, PLK1 localizes to the kinetochore and centrosomes and controls mitotic progression by regulating the spindle assembly checkpoint and attachment of kinetochores to spindle microtubules, which is discussed below. Moreover, PLK1 is involved in regulating other mitosis kinases. PLK1 increases the expression of Aurora B by phosphorylating FoxM1 [74]. PLK1 also activates Haspin by phosphorylation [87,88,89,90,91,92,93,94,95,96,97,98,99,100,101,102,103].

When cells enter anaphase, PLK1 dissipates from the kinetochore, moves to the central spindle and spindle midbody, and plays a role in the initiation of cytokinesis [60,61,63,66,67]. Inhibition of PLK1 abolishes RhoA accumulation at the equatorial cortex and disrupts the localization of Ect2, the GEF protein essential for Rho activation, to the spindle midzone [61]. This suggests that PLK1 is critical for the Ect2-RhoA network, which allows proper cleavage furrow and cytokinetic bridge formation, leading to the initiation of cytokinesis.

## 4. Kinetochore Recruitment Mechanism of PLK1

**Figure 2:**
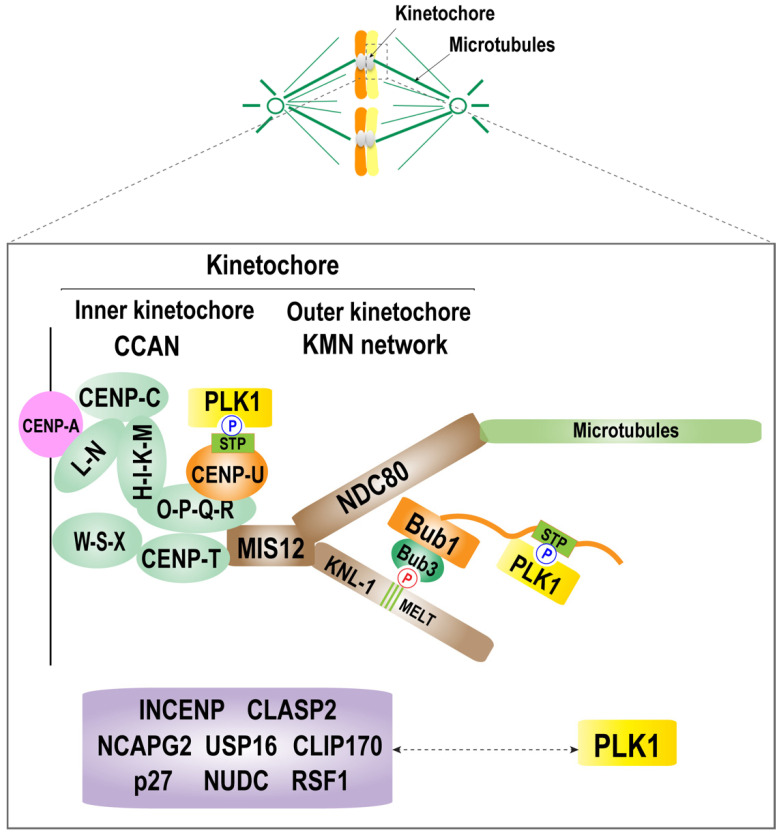
Molecular mechanism of PLK1 localization at the kinetochore. A schematic diagram of the kinetochore structure and the mechanism of kinetochore recruitment of PLK1. The kinetochore consists of an inner and an outer kinetochore. The inner kinetochore is composed of a complex of CCAN, CENP-C, CENP-HIK-LN, CENP-T, and CENP-QU. The outer kinetochore is composed of KNL1 complex, Mis12 complex, and the NDC80 complex (KMN network). PLK1 is recruited to the kinetochore by interacting with CENP-U and Bub1, which are located in the inner kinetochore and outer kinetochore, respectively. There are other known PLK1 receptors, including the kinetochore components Bub1, BubR1, and CENP-U (also called PBIP1), and other proteins, including nuclear distribution protein C (NUDC), CLIP-170, dynactin subunit p27, INCENP, CLASP2, Survivin, NCAPG2, USP16, and RSF1. The relationship between PLK1 and these components during mitosis requires further characterization.

Because of the importance of PLK1 during mitosis, the molecular mechanism of the kinetochore localization of PLK1 has been extensively studied. PLK1 has been suggested to be recruited to the kinetochore by interacting with many proteins, including the kinetochore components Bub1, BubR1, CENP-U (also called PBIP1), and other proteins, including nuclear distribution protein C (NUDC), CLIP-170, dynactin subunit p27, INCENP, CLASP2, Survivin, SGO1, NCAPG2, USP16, and RSF1 [87,88,90,93,94,95,97,98,99,100,101,102,103,104,105,106,107,108]. Localization of PLK1 is mediated by the interaction of its PBD with proteins that contain its S-[pT/pS]-P/X consensus-binding motif, which is primed by the master mitotic kinase Cdk1 [45,46]. Accordingly, all the listed proteins, except SGO1, contain the S-[pT/pS]-P/X motif, and inhibition of these proteins or the introduction of mutations in the S-[pT/pS]-P/X motif results in reduced kinetochore recruitment of PLK1 [87,90,93,94,95,96,97,100,101,102,103,104,106,108] (Figure 2). However, it is unclear whether these interacting partners equally contribute to kinetochore recruitment of PLK1 or whether the initial interaction of PLK1 with only one or few proteins facilitates the interaction of PLK1 with other PLK1-binding proteins.

Additionally, Aurora B may contribute to PLK1 kinetochore localization. Aurora B contributes to the kinetochore localization of Bub1, a kinetochore component that recruits PLK1 to the kinetochore [109]. Moreover, the T-loop in the kinase domain of PLK1 can be phosphorylated by Aurora A or B, resulting in the activation and kinetochore recruitment of PLK1 [50,51]. Furthermore, because Aurora B and phosphatase PP2A-B56 counteract each other during mitosis, Aurora B indirectly inhibits dephosphorylation by PP2A-B56, which in turn contributes to the recruitment of PLK1 at the kinetochore [5,110].

Although several proteins were suggested to recruit PLK1 to the kinetochore, three groups recently suggested that CENP-U and Bub1 are the main PLK1 kinetochore receptors during mitosis [90,104,111]. Nguyen et al. and Chen et al. suggested that Bub1 and CENP-U act in parallel pathways to recruit PLK1 to the kinetochore using cell biological approaches. They both showed that depletion of the CENP-O complex, which includes CENP-U or CENP-U, resulted in a significant decrease in kinetochore localization of PLK1. In addition, depletion of only the CENP-U or CENP-O complex compromised chromosome alignment marginally; however, when it was combined with Bub1 depletion, the PLK1 level at the kinetochore was further decreased, and the chromosome alignment defect was significantly increased. These results indicate that both CENP-U and Bub1 work together to reach a certain threshold level for PLK1 at the kinetochore for normal function. Interestingly, Nguyen et al. showed that the requirement of the CENP-O complex for kinetochore localization of PLK1 was variable between cell lines, which suggests that different expression levels of Bub1 and CENP-U could affect the requirement of these components for normal kinetochore recruitment of PLK1.

Singh et al. first tested the role of Aurora B in recruiting PLK1 to the kinetochore. They showed that the contribution of Aurora B to the PLK1 kinetochore is indirect. Inhibition of Aurora B with hesperadin caused a strong decrease in the PLK1 level at the kinetochore. However, this was rescued with phosphatase inhibitor okadaic acid (OA), suggesting that Aurora B indirectly contributes to the PLK1 level at the kinetochore by counteracting the phosphatases PP2A-B56 and PP1. In contrast, Bub1 directly interacts with PLK1-PBD but only in the presence of priming kinases PLK1 and CDK1 in vitro. Although inhibition of kinetochore recruitment of Bub1 by treating human cells with an Mps1 kinase inhibitor significantly decreased the levels of PLK1 at the kinetochore, it left residual levels of PLK1 at the kinetochore, indicating that additional PLK1 receptors were required. Through in vitro reconstitution of the 22 kinetochore components, Singh et al. found that among the inner kinetochore components that comprise constitutive centromere-associated network (CCAN), CENP-U is the primary receptor of PLK1. In addition, ectopic localization of CENP-U increased the PLK1 level at the ectopic site, suggesting that CENP-U is necessary for the kinetochore localization of PLK1. Furthermore, the co-depletion of Bub1 and CENP-OPQUR complex completely abolished the kinetochore recruitment of PLK1, and the PLK1 level was not rescued by OA treatment, indicating that Bub1 and CENP-U are the main receptors for PLK1 at the kinetochore.

As suggested for other PLK1 receptors, interactions between CENP-U, Bub1, and PLK1 are heavily dependent on priming by CDK1-mediated phosphorylation. Although these results indicate that Bub1 and CENP-U are essential for kinetochore recruitment of PLK1, they do not exclude the possibility that other known PLK1 receptors are important in the normal cell progression because all experiments with human cells are conducted using the nocodazole treatment, which removes the spindle microtubules during mitosis.

Once all kinetochores attach to the spindle microtubules at metaphase, PLK1 is ubiquitinated by active APC/C, resulting in its dissociation. Ubiquitin-specific peptidase 16 (Usp16) antagonizes the activity of CUL-3-based ubiquitin ligase by deubiquitinating PLK1 at the kinetochore [94]. Furthermore, Usp16 promotes chromosome alignment by regulating kinetochore localization of PLK1 through phosphorylation of the Cdk1 site of USP16 [94].

In the future, it will be valuable to investigate whether the initial recruitment of PLK1 by CENP-U and Bub1 activates phosphorylation of downstream targets that promote further recruitment of PLK1, thereby clarifying how the PLK1-binding proteins at the kinetochore regulate PLK1 dynamics and function.

## 5. PLK1 Function in Spindle Checkpoint Activation and Spindle Checkpoint Silencing

Although the kinetochore localization of PLK1 increases at metaphase, how PLK1 functions within the kinetochore is unclear because of the complexity of the interdependence of many kinases and phosphatases and because many PLK1 receptors are distributed along the kinetochore and centromere.

One of the major functions of PLK1 during mitosis is the activation of the spindle assembly checkpoint (SAC). The SAC is a safeguard system that halts anaphase progression by inhibiting APC/C until all kinetochores attach to the spindle microtubules. APC/C inhibition is caused by the accumulation of SAC components at unattached kinetochores, a process that mediates the formation of the mitotic checkpoint complex (MCC) composed of Mad2, Bub3, BubR1, and Cdc20 [3,112].

The accumulation of SAC components involves phosphorylation of SAC components by Mps1 kinase [113,114,115,116,117,118,119,120,121,122]. Both PLK1 and Mps1 share a similar phosphorylation consensus motif D/E-x-S/T-Φ, with a strong preference for D/E residues at position-2 [123,124,125,126,127]. Consequently, PLK1 and Mps1 may share many SAC substrates. *Caenorhabditis elegans* lacks the Mps1 kinase; thus, PLK1 potentially phosphorylates several known Mps1 substrates, which is discussed later [128]. However, it remains unclear whether PLK1 can completely substitute for Mps1 kinase in other animal systems.

The Bub1/Bub3 complex activates SAC by recruiting Mad1, Mad2, BubR1, and Cdc20 to unattached kinetochores. Kinetochore recruitment of Bub/Bub3 is mediated by the core outer kinetochore component KNL1 via multiple “MELT (Met-Glu-Leu-Thr)” motifs, which are phosphorylated by Mps1 and PLK1 [113,114,115] Additionally, Mps1 phosphorylates the CM1 motif of Bub1, and its phosphorylation is required for its interaction with Mad1 [129,130]. Alternatively, PLK1 is able to phosphorylate the Bub1-CM1 motif in *C. elegans* [89]. Mps1 is also suggested to phosphorylate MAD-1 C-terminus to promote interaction with the N-terminal region of CDC20 [129,131]. In vitro data using *C. elegans* proteins showed that phosphorylation of PLK-1 at the MAD-1 C-terminus promoted its interaction with the CDC-20 N-terminus [89].

PLK1 also contributes to SAC activation by phosphorylating the APC/C co-activator Cdc20. PLK1 that interacts with S-[pS/pT]-[P/X] motif of Bub1 at the kinetochore suggested to phosphorylate Cdc20, resulting in the inhibition of the interaction between Cdc20 and APC/C [132].

**Figure 3:**
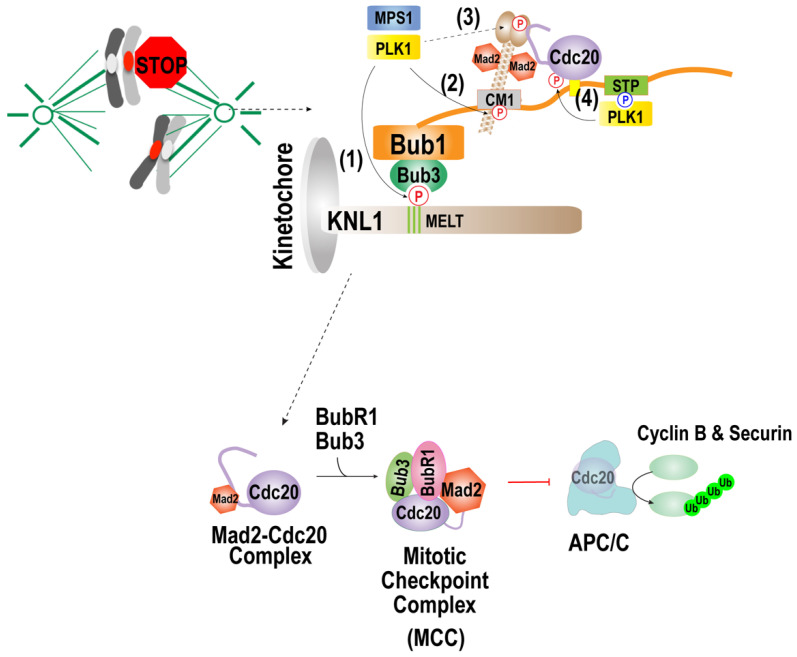
Function of the PLK1 in spindle assembly checkpoint activation. Reprinted/adapted with permission from Ref. [133], 2021, Taekyung Kim. When kinetochores are not attached to the microtubules, PLK1 activates the checkpoint by phosphorylating (1) MELT motifs of KNL1 and (2) the CM1 motif of Bub1, (3) C-terminal region of Mad1, and (4) Cdc20. The PLK1 can substitute Mps1 kinase in phosphorylating MELT motifs of KNL1, which promotes kinetochore localization of the Bub1/Bub3 complex and the CM1 motif of bub1, mediating the interaction with Mad1. PLK-1 in *C. elegans* was shown to phosphorylate the C-terminal region of Mad1, which promotes the interaction with Mad1. PLK1 that interacts with Bub1 activates the MCC formation by phosphorylating Cdc20.

Collectively, these results suggest that PLK1 may activate SAC by phosphorylating Knl1, Bub1, Mad1, and Cdc20. When kinetochores are unattached, phosphorylation-mediated interaction of Cdc20 with Bub1 and Mad1 can occur at the kinetochores, which is critical for the formation of Mad2-Cdc20, which mediates the formation of MCC (Figure 3). However, which pool of PLK1 phosphorylates these kinetochore components in vivo requires further investigation. It will be enlightening to knock down specific PLK1 pools that interact with Bub1 or CENP-U, or other known PLK1 receptors, to determine the effects of the phosphorylation of one of those PLK1 substrates and the SAC activation.

In addition to substituting for Mps1 activity, PLK1 activates Mps1 by phosphorylating multiple sites of Mps1, although whether such phosphorylation directly activates Mps1 is unclear [134].

Once all kinetochores are attached to spindle microtubules, all SAC components are removed from the kinetochore, which extinguishes the SAC signal and anaphase progression by activating APC/C [3]. For SAC silencing, PP2A-B56 and PP1 are the major phosphatases that dephosphorylate the MELT motifs of KNL1, thereby removing Bub1 from the kinetochore [135,136,137,138,139,140,141,142,143]. Although PLK1 may activate SAC, paradoxically, PLK1 may also help silence SAC by recruiting PP2A to the kinetochore. BubR1 contains a KARD motif that interacts with PP2A-B56, and phosphorylation of the KARD motif by PLK1 enhances the interaction with PP2A-B56 [99,144,145]. However, PP2A-B56 and PP1 may silence the spindle checkpoint by dephosphorylating the polo-binding motif of Bub1 and BubR1, which removes PLK1 from the kinetochore [146]. To clarify the function of PLK1 in the SAC, the functions of phosphatases PP1 and PP2A-B56 in SAC silencing must be studied. Whether the removal of PLK1 from the kinetochore is the major cause of silencing of the spindle checkpoint requires further study.

## 6. A Role of PLK1 in Stabilizing Kinetochore-Microtubule Attachment

In addition to the function of PLK1 in SAC activation, PLK1 plays a role in stabilizing the kinetochore-microtubule attachment during prometaphase [62]. This is somewhat contradictory because SAC activation delays the onset of anaphase, and stabilizing kinetochore-microtubule attachment promotes the onset of anaphase. Mps1, which can be substituted by PLK1 in some cases, activates the SAC and inhibits the kinetochore-microtubule attachment. Inhibition of PLK1 can destabilize or stabilize kinetochore-microtubule attachment under different experimental conditions [55,77,110]. Potentially, PLK1 initially helps form the kinetochore-microtubule attachment by activating the SAC, and once the kinetochore interacts with the microtubule, PLK1 helps stabilize the kinetochore-microtubule attachment. The dual function of the PLK1 may help cells respond quickly and efficiently to the change in the kinetochore-microtubule dynamics. However, whether or how PLK1 activity is controlled within such a short period needs to be further studied.

Among all suggested PLK1 receptors that localize at the kinetochore or centromere, some may contribute to stabilizing kinetochore-microtubule attachment. CENP-U, BubR1, PICH, CLIP-170, CLASP2, Survivin, USP16, and RSF1, which interact with PBD and are phosphorylated by PLK1, showed defects in chromosome alignment when a phospho-mutant was introduced [62,90,92,94,96,97,99,100,105,106,136,144,145,147]. However, it is unclear whether the defects in the phospho-mutants of PLK1 receptors are caused by reduced localization of PLK1 or other functions of PLK1 receptors. The localization of PLK1 in a specific region by PLK1 receptors may promote phosphorylation of other proteins at close distances that participate in kinetochore-microtubule attachment. For instance, the kinetochore-localized pool of PLK1 binds to CENP-U phosphorylates CENP-Q at multiple sites; mutations of these phosphorylation sites can cause defects in chromosome segregation [148,149]. PLK1 also contributes to the recruitment and phosphorylation of Sgt1, a co-chaperone for Hsp90 [101]. Hsp90 stabilizes the kinetochore complex by interacting with the MIS12 complex, thereby coordinating the recruitment of NDC80, which interacts with spindle microtubules [150]. In addition, PLK1 interacts with and phosphorylates astrin, which is essential for chromosome alignment and microtubule attachment [151].

Finally, PLK1 may promote stable kinetochore-microtubule attachment by controlling the pool of kinases and phosphatases. As described earlier, BubR1 contains a KARD motif that recruits PP2A, which promotes stable kinetochore-microtubule attachment by counteracting Aurora B activity. PLK1 enhances PP2A interactions by phosphorylating the KARD motif [99,144,145]. However, other studies suggest that PLK1 activates Aurora B by phosphorylating the activating loop of Aurora B [88]. Furthermore, Aurora B may contribute to kinetochore localization of PLK1 and activate PLK1 by phosphorylating the activation loop in the kinase domain [152]. Therefore, regulation of the balance of the phosphorylation state by PLK1 is complex and remains to be fully determined.

## 7. Concluding Remarks

Since the discovery of PLK1 in the early 1980s, it has been extensively studied using structural, biochemical, and cell-biological approaches. PLK1 contains a conserved kinase domain and PBD domains that recognize the S-[pS/pT]-[P/X] motif of the target protein, required for localization and activation. PLK1 is a master kinase with diverse functions in mitosis. PLK1 localizes to the specific regions in cells through interaction with many proteins, including the PBD domain, and locally phosphorylates the substrates to perform diverse functions. How PLK1 localizes at the kinetochore and regulates functions in mitosis is an important question to be resolved. Many kinetochore proteins, which contain a motif that interacts with the PBD domain, have been suggested to recruit PLK1 to the kinetochore. Here, we reviewed the recent studies that suggest the primary receptors of the PLK1 at the kinetochore. Intriguingly, CENP-U and Bub1 are suggested to be the primary receptors of the PLK1 at the kinetochore, although the relationship between PLK1 and other known PLK1 receptors remains to be clarified. PLK1 contributes to SAC by phosphorylating Knl1, Bub1, BubR1, Mad1, Cdc20, and Mps1. In addition, PLK1 contributes to kinetochore-microtubule attachment via multiple pathways involving PLK1 phosphorylation. Significant progress has been recently made, although many important questions remain. For example, understanding the temporal regulation of PLK1 during mitosis is important. How PLK1 and other kinases, Mps1 and Aurora B, and phosphatases, PP1 and PP2A, work together to balance the phosphorylation state of the kinetochore proteins involved in mitosis remains unresolved. Additionally, since PLK1 localizes at the kinetochore, the kinetochore-localized pool of PLK1 might phosphorylate all those substrates involved in the SAC activation and the kinetochore-microtubule attachment. However, whether or when the kinetochore-localized pool of PLK1 phosphorylates these substrates needs to be investigated. Finally, the identification of additional substrates of PLK1 during SAC activation and normal cell cycle progression will herald the development of new approaches for treating diseases such as cancer.

## Data Availability

Not applicable.
